# Effectiveness of Ethnic-Cultural Educational Strategies for the Promotion of Breastfeeding

**DOI:** 10.3390/ijerph22091416

**Published:** 2025-09-11

**Authors:** José Fabián Hidrobo-Guzmán, Gladys Edelmira Morejón-Jácome, Edison Daniel Cárdenas-Robles, Lizbeth Dayana Pilco-Vargas, Doménica Vanesa Posso López, Estefany Tatiana Iguago Angamarca

**Affiliations:** Faculty of Health Sciences, Technical University of the North, Ibarra 100150, Ecuador; gemorejon@utn.edu.ec (G.E.M.-J.); edcardenasr@utn.edu.ec (E.D.C.-R.); ldpilcov@utn.edu.ec (L.D.P.-V.); dvpossol@utn.edu.ec (D.V.P.L.); etiguagoa@utn.edu.ec (E.T.I.A.)

**Keywords:** educational intervention, breastfeeding, ethnic-cultural, prenatal stage

## Abstract

**Introduction:** The implementation of pedagogical tactics with ethnic-cultural approaches represents essential mechanisms for promoting breastfeeding, ensuring greater effectiveness in knowledge dissemination. These are supported by cultural ideologies that integrate didactic processes tailored to the beliefs and practices of each ethnic group, thus facilitating greater acceptance and adherence to health recommendations. **Objective:** The study aims to analyze the effectiveness of culturally sensitive educational strategies from the prenatal stage at the Quiroga Health Center, Ecuador, exploring how indigenous cultural dynamics influence the receptivity of education. **Methodology:** The research is quantitative, quasi-experimental, and employs a descriptive, documentary, bibliographic, and analytical cross-sectional cohort design to thoroughly analyze collected data and identify cause-and-effect factors influencing breastfeeding knowledge and practice. **Results:** The effectiveness of the educational intervention in promoting cognitive awareness of breastfeeding was confirmed using the McNemar statistic applied to related samples. **Conclusions:** Breastfeeding knowledge levels are closely tied to reproductive history, a constantly evolving process affected by various social factors. Health education is implemented and strengthened through teaching programs as these strategies can significantly contribute to maternal and child health promotion, adapting to the specific needs and contexts of each community.

## 1. Introduction

Health education interventions play a crucial role in strengthening knowledge and sanitary practices. En Guatemala el Census-Based, Impact-Oriented (CBIO) demonstrated significant improvements in maternal and child health indicators by expanding coverage through community-based interventions. This approach involved training women volunteers to deliver health education, which resulted in marked improvements in knowledge and practices related to maternal and newborn health, nutrition, and disease prevention [1,2].

In Ghana, community health interventions led to increased antenatal and postnatal care coverage, higher rates of facility-based deliveries, and improved treatment of childhood illnesses. These interventions empowered community members and improved health system performance, showcasing the effectiveness of community engagement in health education [3].

Health education contributes to reducing health inequities, improving maternal and child health indicators, and expanding coverage to vulnerable populations [4].

Culturally adapted educational approaches improve the dissemination of health information. By respecting local customs and beliefs, they help build trust with pregnant women, increasing their engagement and self-esteem [5].

Transcultural breastfeeding education aims to prolong breastfeeding practices. Breast milk provides immune protection, supports neurological development, and offers benefits to both mother and child [6].

Universal education transforms the world, grounded in a process based on the traditions, customs, and beliefs of diverse social groups, regardless of their ethnic origin [7]. According to UNICEF, breastfeeding educational interventions should be culturally delivered from the primary care level as they facilitate and improve misconceptions that hinder the practice through pedagogical tactics that reduce maternal and child mortality rates [8].

In 2019, approximately 5.2 million children under five died due to inequalities in access to healthcare caused by a lack of education and information, with over half being newborns [9]. Statistical contributions from the Pan American Health Organization (PAHO) highlight that globally, only about 50% of children are breastfed within the first hour after birth, and just 32% of children under six months are exclusively breastfed [10]. These figures vary due to cultural factors specific to each ethnicity, as deeply rooted beliefs prevent initiating and maintaining exclusive breastfeeding. For example, some cultures consider the first milk (colostrum) to be harmful to newborns [11].

In Ecuador, the Ministry of Public Health promotes breastfeeding through programs and regulations such as ESAMyN (Mother and Child-Friendly Health Facilities), which focus on protecting and supporting breastfeeding. These initiatives are strengthened by the creation of groups composed of pregnant women and healthcare professionals in health centers across the country. These programs aim to improve pregnant women’s knowledge, attitudes, and practices related to breastfeeding. By offering respectful, continuous, and culturally appropriate care, they support a healthy start to life while aligning with constitutional rights and Ecuador’s Comprehensive Health Care Model to reduce maternal and child mortality [12,13].

The main health programs and projects in Ecuador focus on promoting and educating pregnant women about the benefits of breastfeeding during prenatal checkups, making this practice a culturally essential experience passed down through generations to combat chronic childhood malnutrition [14]. The 2018 National Health and Nutrition Survey revealed that early breastfeeding initiation (within the first hour of birth) reached 72.7%, a significant increase from the 54.4% reported in 2014 [15], differences entirely framed by the results of educational interventions adopted in the health sector [16].

Nola Pender’s conceptual model and Dorothy Johnson’s Behavioral Theory, pioneers in health promotion applied to populations with disorganized, irregular, and dysfunctional behaviors that affect physical, mental, and social well-being, guide the World Health Organization (WHO) in emphasizing the critical role of nurses as the first direct link between the healthcare system and users. Their goal is to improve patients’ lifestyles. In this sense, breastfeeding educational strategies are designed to correct and reinforce misconceptions among pregnant women while respecting their cultural doctrines [17].

Ramona Mercer’s theories on Maternal Role Attainment and Madeleine Leininger’s Transcultural Care highlight the importance of nursing staff in patient care. Mercer asserts that the role of these professionals in family contexts and couple dynamics strengthens the emotional bond between mother and child, primarily influenced by cultural customs. Leininger, with her ethnonursing approach, emphasizes addressing healthcare aligned with people’s beliefs, practices, and lifestyles to qualitatively identify the internal perspectives of communities [17].

This research aims to analyze the effectiveness of educational strategies with an ethnic-cultural focus to promote breastfeeding among pregnant women attending the Quiroga Health Center. Its significance lies in educating and empowering pregnant women as the cornerstone for raising healthy, strong children by providing timely education that enables a comprehensive understanding of the topic from their cultural worldview. To this end, an initial assessment was conducted to determine prior knowledge levels, followed by the implementation of tailored strategies for the selected population. Subsequently, the effectiveness of these educational actions was evaluated through a final assessment to confirm increased knowledge. Finally, factors influencing the population’s perception were analyzed.

The study has a positive economic and social impact, as promoting breastfeeding prevents maternal and child illnesses, reducing healthcare costs by avoiding expenses on medical services and infant formulas, thus improving family cohesion. Therefore, children and mothers are the primary beneficiaries. Studies suggest that breastfed infants achieve better learning capacity and academic performance, while mothers experience faster postpartum recovery and reduced risk of postpartum depression [18].

The scope of the results focused on determining whether the prenatal educational process defined behavioral patterns linked to cultural identity, including preferred educational strategies such as talks, videos, games, dramatizations, and group discussions, supported by didactic and printed materials to reinforce teaching frameworks that promote appropriate breastfeeding techniques from birth to two years of age.

Studies conducted in São Paulo, Brazil, reveal that health education interventions benefit, promote, and prolong breastfeeding. These strategies provide verbal, visual, and tactile stimuli that enhance understanding and effective learning for good practices [19]. Research in Latin America and the Caribbean indicates that breastfeeding, as an essential practice, strengthens the newborn’s immune system, reducing future risks of obesity and type 2 diabetes [10].

Breast milk is the ideal food for children, containing antibodies, proteins, carbohydrates, lipids, vitamins, and other nutrients that positively regulate growth and development in early childhood, especially in the first six months [20]. It occurs in three stages: colostrum, produced up to 72 h postpartum, is dense and yellowish, with a high concentration of immunoglobulins and amino acids that aid digestion and expulsion of the first stools (meconium), justifying its intake within the first 30 min after birth. Transitional milk, produced from days four to ten, is creamier with higher fat, lactose, calorie, and water-soluble vitamin content than colostrum. Mature milk, the final stage, is more diluted and bluish, rich in fat-soluble proteins, vitamins E, A, K, and minerals like sodium, zinc, iron, sulfur, and potassium [21,22].

Breastfeeding is a wonderful experience that should not be painful. However, issues like mastitis, inverted nipples, cracked nipples, or blocked ducts can cause pain, swelling, and irritation, often due to improper latch or lack of stimulation. Preventive measures in breastfeeding techniques are crucial, involving instructions for successful breastfeeding. Proper breastfeeding technique involves not only ensuring the mother’s comfort—whether sitting or lying down—but also correct positioning of the baby. This includes aligning the baby’s nose with the nipple, maintaining belly-to-belly contact, supporting the baby’s back and head, and holding the breast in a C-shape. The nipple should gently stimulate the baby’s mouth to encourage a deep latch that covers the entire areola [23,24]. Another important advancement in breastfeeding promotion is the establishment of milk banks—hospital-based centers that provide donated breast milk to infants who cannot be breastfed by their mothers [25].

This initiative led to home milk banking, involving milk extraction when mother and child are apart. Safe procedures include rigorous hand hygiene and proper extraction techniques, using a C-shape grip behind the areola to collect milk in glass containers or bags, labeling with date and time, and storing in the refrigerator. To feed the child, thawing via the bain-marie method preserves nutrients [26].

In healthcare, education plays a vital role in fostering healthy behaviors to address issues and improve quality of life. Educational strategies, applied in various social contexts, influence practices and knowledge. Direct strategies, like educational talks, videos, didactic games, and dramatizations, focus on interaction and language to aid memorization and comprehension. Indirect strategies, like brochures and leaflets, allow recipients to interpret information independently, promoting authentic understanding. These resources, printed or digital, are designed to present relevant information concisely to capture attention and promote learning [27].

Health education interventions are didactic and pedagogical processes that provide information and teaching to improve knowledge, skills, and behaviors at individual and community levels. Specifically, breastfeeding promotion strategies are implemented and reinforced through pedagogical interventions focused on health, grounded in the customs and beliefs of various ethnic groups, emphasizing pregnant mothers. This education facilitates the transmission of greater knowledge, impacting early breastfeeding practices, benefiting mother and child at birth and beyond by preventing early abandonment, aligning with the bioethical principle of beneficence to enhance patient well-being and prevent adverse health impacts.

## 2. Materials and Methods

To achieve the proposed goals, the study employed a quantitative research methodology to analyze and quantify issues related to breastfeeding knowledge, practices, and attitudes, establishing objective and systematic cause-and-effect relationships. It is quasi-experimental, evaluating interactions randomly in practical settings without intentionally manipulating variables, using interventions that yield ethically acceptable outcomes. The analysis was based on a descriptive and analytical cross-sectional cohort design, incorporating documentary and bibliographic approaches, with detailed explanations of the characteristics of individuals, situations, and groups, collected for examination using statistical techniques to provide clear, understandable data within the established timeframe and related to the prevalence of behaviors surrounding the issue, ensuring content reliability [28].

The research was conducted at the Quiroga Health Center, a rural parish in the Cotacachi canton, Imbabura province, Ecuador’s Zone 1, Health District 10D03. The area has approximately 7489 inhabitants, including mestizos, whites, and indigenous people across 11 communities. The target population consisted of 109 pregnant women attending prenatal checkups at the health facility from November 2023 to February 2024, included only if they consented to participate via informed consent. The sample size of 109 participants was determined through non-probabilistic convenience sampling, based on the total number of pregnant women who attended prenatal checkups at the Quiroga Health Center during the specified period and voluntarily agreed to participate in the educational intervention. Given the quasi-experimental nature of the study, with a cross-sectional cohort design and no intentional manipulation of variables, a formal a priori power calculation was not conducted. However, the number of participants was sufficient to detect statistically significant differences using the McNemar test for related samples (*p* < 0.001), confirming the adequacy of the sample size to evaluate the effectiveness of the educational strategies applied.

The questionnaire was designed collaboratively by the research team, including faculty members and research assistants from the Faculty of Health Sciences at Universidad Técnica del Norte. Its development was grounded in a comprehensive review of scientific literature and national health education guidelines related to breastfeeding promotion. To ensure content validity, the instrument underwent expert evaluation by three professionals in maternal and child health and intercultural health education, who assessed the relevance, clarity, and cultural appropriateness of each item. Based on their feedback, modifications were made to optimize the instrument’s structure and comprehension.

A pilot test was conducted with a group of 15 pregnant women from a neighboring health center not included in the final sample, to verify the clarity and applicability of the questions in a real-world setting. No major difficulties were reported. To assess the internal consistency of the instrument, the Kuder–Richardson Formula 20 (KR-20) was applied, and given the dichotomous nature of the items, the obtained reliability coefficient of 0.76 indicates acceptable reliability.

The survey was applied via the QuestionPro platform in two stages: the first measured sociodemographic and obstetric data, educational promoters, cultural factors, pedagogical strategy preferences, and prior knowledge (pretest) with 41 questions. The second, with 16 questions, reassessed knowledge post-intervention (posttest) to evaluate the effectiveness of the applied strategies.

The collected data were used to create an Excel database, later exported to IBM SPSS version 25, a program offering statistical analysis tools for effective data exploration and visualization. The McNemar hypothesis test was used to measure intervention effectiveness, identifying significant differences by comparing pre- and post-intervention results from related samples across two time periods, assessing improvements in knowledge and behavior regarding traditional ideological stances expressed by pregnant women [29].

Based on the initial diagnosis, learning needs were identified, and educational talks with PowerPoint presentations covered topics like breastfeeding definitions, breast milk composition, benefits, breast conditions, breastfeeding techniques, and milk banks. Videos, interactive question-and-answer activities with dice and cards, dramatizations using anatomical simulators, and group discussions about customs, traditions, ideologies, and experiences were conducted. These activities were supported by distributed brochures, leaflets, and flyers detailing the information, held over six months.

## 3. Results and Discussion

Table 1 presents the baseline sociodemographic characteristics of the 109 pregnant women who participated in the study. Most respondents were aged between 21 and 35 years (68.8%), belonged primarily to the indigenous (50.5%) and mestizo (46.8%) ethnic groups, and were housewives (71.6%). In terms of education, the majority had completed secondary education (43.1%), while 12.8% had only primary education, and 6.4% reported no formal education. These baseline characteristics are relevant in understanding the context and potential influencing factors on breastfeeding knowledge and practices.

The tables and results below describe the behavior of the studied population regarding the effectiveness of ethnic-cultural educational strategies for breastfeeding promotion at the Quiroga Health Center, 2023–2024, achieved through data processing, analysis, and discussion per the research’s purpose and methodology. The Richardson statistic yielded a 76.0% reliability index for the data collection tool, indicating an acceptable confidence level. The Kolmogorov–Smirnov normality test confirmed that quantitative and ordinal variables did not follow a normal distribution, necessitating non-parametric tests for statistical analysis.

Table 2 presents the 2 × 2 contingency table comparing participants’ breastfeeding knowledge before and after the educational intervention. Among the 79 pregnant women who initially lacked knowledge, 35 showed improvement after the intervention. Notably, all 30 participants who already had knowledge retained it. This distribution indicates a positive shift in awareness.

The McNemar test was applied to assess whether this change was statistically significant in paired samples. The result showed a test value of 2.28 with a bilateral exact significance of *p* = 0.000 (<0.05), indicating a statistically significant improvement in knowledge following the intervention.

However, this result must be interpreted with caution. The McNemar test is a bivariate method that evaluates only the change between two related categorical variables and does not control for potential confounding variables, such as age, education, or ethnicity. Therefore, while the intervention appears to have had a significant effect, these findings are preliminary. A multivariate analysis (e.g., logistic regression) would be necessary to confirm the independent impact of the educational strategies on knowledge gain, adjusting for relevant sociodemographic characteristics.

Numerous studies confirm that maternal and child educational interventions reduce misinformation and improve breastfeeding practices and attitudes by providing culturally relevant education. These interventions strengthen the ability to discern and clarify misconceptions, thereby developing skills that positively influence behavior and quality of life. Breastfeeding strategies also help strengthen the emotional bond between mother and child, reduce morbidity and mortality, and foster healthy cultural habits [30].

The data in Table 3 show the interrelation between sociodemographic variables and knowledge evaluation. This information indicates that the 21–35 age range constitutes the largest proportion of the studied population, at 68.8%, and these women initially faced the greatest challenges, with an ignorance level of 74.7%, which decreased to 41.3% after the intervention. Regarding knowledge assessed in this range, it increased from 25.3% to 58.7% following participation in the educational activities implemented in the project; however, these results also confirm challenges within this group compared to other age ranges. The analysis of women under 20, representing 17.4% of the respondents, shows an initial ignorance level of 68.4%, which dropped to 36.8% post-intervention. This age category was confirmed to have acquired the most knowledge, rising from 31.6% to 63.2%. The findings in this group suggest a positive impact, as they demonstrate greater competence in assimilating and adapting to new concepts, along with better cognitive flexibility and capacity for learning. This is supported by technological components that enable comprehensive development, allowing them to engage virtually with educational resources freely, timely, privately, and independently in a context of personal empowerment for decision-making and forming health habits [31].

Regarding marital status, the largest group of women studied (43.1%), who initially showed an ignorance level of 59.6% that later decreased to 34.0% and increased their knowledge from 40.4% to 66.0%, were married. In contrast, those in free unions (22.9%) revealed a prior ignorance level of 88.0%, which dropped to 50.9% post-evaluation, showing an increase in learning from 12.0% to 64.0%. Comparing these results, the training was more effective for those in free unions, primarily due to greater decisional autonomy and less social pressure in more flexible, comfortable, and safe households for learning without prejudice [32].

The most prevalent ethnicity studied was indigenous (50.5%), settled in rural areas (53.8%), who initially showed an ignorance level of 80.0%, which decreased to 50.9%, resulting in an increase in knowledge from 20.0% to 49.1%. In contrast, the mestizo group (46.8%) reduced their ignorance from 62.7% to 27.5%, better assimilating the applied didactic teaching methods, as their knowledge increased from 37.3% to 72.5%. This outcome reflects the country’s current socioeconomic conditions, supporting the mestizo sector with greater access to education and fewer collective and financial difficulties in a cultural context that favors information perception and processing. Conversely, the indigenous group faces limited maternal education opportunities due to language barriers and racial and gender discrimination prejudices, which hinder communication and comprehension [33].

To determine whether the observed differences in knowledge before and after the intervention were statistically significant across sociodemographic groups, the Chi-square test was applied. Statistically significant associations (*p* < 0.05) were found for age group, marital status, ethnicity, and education level, suggesting these variables had an influence on knowledge gain. Although occupation showed a trend toward significance, it did not reach the threshold. These results highlight the relevance of contextual sociodemographic factors when designing and delivering culturally sensitive health education strategies.

Most participants were housewives (71.6%) and part of nuclear families (54.1%), initially showing an ignorance level of 74.4%, which decreased to 38.5%, with knowledge increasing from 25.6% to 61.5%. Notably, the intervention had a greater impact on students (8.3%), who, despite an initial ignorance level of 77.8%, ended with 33.3%, leading to a knowledge increase from 22.2% to 66.7%. Statistics indicate that these women have higher educational levels, reading skills, and social interaction, enabling more effective comprehension and analysis of received information. This distinguishes them from housewives, who, due to household obligations, face significant challenges in meeting their children’s needs in early years, stemming from lower formal education and fewer opportunities to engage in discussions, leading to neglect of their autonomy and responsibility for childcare [34].

Regarding educational level, 43.1% of respondents had secondary education, showing an initial ignorance level of 63.8% and a final level of 34.0%, with knowledge progressing from 36.2% to 66.0%. In contrast, those with higher education (11.9%) initially showed an ignorance level of 38.5%, which decreased to 15.4% post-intervention, standing out for the greatest knowledge increase, from 61.5% to 84.6%. These percentages are fully justified by studies confirming that higher education fosters more developed cognitive skills for understanding and retaining content with positive learning attitudes in a supportive social and family environment. In contrast, those with secondary education experience a lack of supplementary maternal information related to their age [35].

The obstetric data averages from the survey are as follows: 2.25 pregnancies, 1.70 vaginal deliveries, 0.31 cesareans, and 0.24 abortions, with maximums of 8 pregnancies, 7 vaginal deliveries, 2 cesareans, and 2 abortions. These data confirm that most participants were first-time mothers, a situation that fosters their interest in learning about breastfeeding due to the emotions and feelings associated with first-time motherhood, coupled with an unconditional desire to ensure the newborn’s well-being. This contrasts with experienced mothers who show less motivation to learn [36].

Table 4 summarizes the sources of educational support that participants reported as influential in acquiring breastfeeding knowledge. This directly supports the research objective by identifying which community actors or institutions are most effective in disseminating health education. The majority of participants (33.3%) reported that healthcare personnel—primarily nurses and obstetricians—played a key role in guiding and reinforcing breastfeeding practices during prenatal visits. Family members (27.9%) and women in the community (12.2%) also had a notable influence, highlighting the importance of incorporating local social structures and cultural familiarity into the design of educational strategies. These findings emphasize the need for interventions that actively involve culturally trusted figures to enhance message reception and adherence. The shared stories from these groups underscore the importance of accompaniment (a direct communication link with the family) and the counseling role fulfilled by nurses at the primary care level [37,38].

It can be conjectured that, throughout Ecuadorian history, the customs, traditions, and myths incorporated by different cultures have affected breastfeeding knowledge and attitudes, leading to early abandonment of this practice, which negatively impacts key perinatal indicators. Table 5 presents the most frequently cited cultural beliefs and perceptions that contribute to the abandonment or early interruption of breastfeeding. This analysis is fundamental to the study’s objective as it identifies sociocultural barriers that may hinder the success of educational interventions. The most prevalent belief, reported in 31.4% of responses, relates to stigmas such as shame around public breastfeeding, misconceptions about colostrum, and perceived maternal inadequacy. This category includes traditional paradigms such as the inappropriate behavior of breastfeeding in public, the return of menstruation, and perceived maternal incompetence due to low milk production [39]. Other factors include community and personal pressure (26.3%), such as balancing work or education responsibilities, and traditional views that promote early weaning or associate breastfeeding with undesirable outcomes. These cultural constructs must be acknowledged and addressed within educational programs to ensure culturally respectful and effective health promotion, especially in indigenous and rural settings. To a lesser extent, there is the dietary transition (16.4%) due to a new pregnancy causing early weaning of the child, breast-related conditions (15.1%), and interrupted breastfeeding (10.8%) with the intention of avoiding inappropriate future behaviors (vain women and spoiled men) [40].

Taken together, the results in Table 4 and Table 5 reinforce the importance of designing breastfeeding education programs that are both community-based and culturally responsive. By integrating trusted local actors and addressing deeply held beliefs, interventions are more likely to succeed in promoting sustained breastfeeding practices among diverse populations.

Similarly, the research reveals certain cultural beliefs related to the volume of breast milk, including the consumption of coladas, panela water, yuca, the use of warm cloths, and avoiding exposure to cold, which significantly influence milk production. These practices align with health specialists’ recommendations to consume abundant fluids and to maintain a balanced diet and positive mood, as these factors strengthen prolactin hormone production [41]. Additionally, there are other local customs rooted in the popular knowledge of the evaluated community, notably the belief that, after an episode of maternal anger, the first milk feed should be discarded to prevent colic in the child. This belief, culturally known as “colerín pediátrico,” is thought to potentially cause death. Other beliefs include considering breastfeeding a natural contraceptive, viewing breastfeeding as painful, and deeming colostrum an inadequate food. All these lack scientific basis and stem from erroneous ideas passed down from ancestors to descendants [22].

The findings of this study demonstrate a significant improvement in breastfeeding knowledge among pregnant women following culturally tailored educational interventions. However, beyond the statistical increase, it is essential to interpret the broader meaning of these results within their cultural and regional context. The effectiveness of the intervention reflects not only the educational content but also the alignment of that content with local values, beliefs, and trusted communicators.

In Latin America, previous studies have shown that maternal education alone is often insufficient to change breastfeeding behaviors unless it is culturally relevant and community-based. For instance, a study in rural Mexico by Aquino et al. [24] emphasized that traditional myths—such as viewing colostrum as harmful—can undermine the impact of clinical advice. Similarly, research in Peru and Bolivia has highlighted that women are more receptive to messages delivered by community health workers or elder mothers respected in their culture [22].

These parallels confirm the relevance of our strategy: by involving healthcare professionals and recognizing cultural promoters (e.g., family members, community women), the intervention built trust and legitimacy. Moreover, the improvement in knowledge among younger women and those with higher education may also reflect generational shifts in openness to evidence-based health information, as supported by studies from Brazil and Colombia [19].

Cultural factors had a dual effect: while they enhanced the intervention when respected and incorporated (e.g., using traditional gatherings or oral storytelling), they also posed barriers. As seen in Table 5, stigmas around breastfeeding in public or during menstruation, as well as beliefs about maternal anger affecting milk quality (“colerín pediátrico”), remain deeply embedded. These beliefs are not merely misinformation but part of the cultural worldview, which means they require careful negotiation, not confrontation. Educational strategies that ignore or contradict these perceptions outright are likely to be rejected or ignored.

Therefore, the success of the intervention can be attributed to its culturally congruent delivery—respecting traditions, leveraging community support, and addressing myths with empathy rather than opposition. These findings are consistent with Mercer’s and Leininger’s theories, which emphasize the importance of transcultural nursing and maternal role attainment in health promotion.

In summary, this study supports the growing body of evidence in Latin America that culturally sensitive educational strategies are essential for improving breastfeeding knowledge and practices. Future interventions should further integrate local languages, customs, and intergenerational storytelling methods, especially in indigenous populations where Western biomedical discourse alone may not be persuasive.

## 4. Conclusions

This study demonstrates that culturally tailored educational interventions can significantly improve breastfeeding knowledge among pregnant women in rural and indigenous populations. The success of the strategy lies in its sensitivity to local beliefs, its use of accessible language, and the involvement of trusted community figures such as healthcare personnel and family members.

While quantitative results confirmed the intervention’s effectiveness, qualitative elements—such as respect for traditional practices and addressing cultural myths—were crucial in building trust and engagement. These findings underscore the importance of transcultural approaches in maternal health education, particularly in Latin American settings where cultural identity strongly shapes health behavior.

Public health policies should prioritize community-based strategies that incorporate cultural promoters and address sociocultural barriers to breastfeeding. Future research should explore the long-term effects of such interventions and assess their scalability in diverse regional contexts using multivariate models to isolate the influence of sociodemographic factors.

In conclusion, integrating cultural knowledge into health education is not only respectful—it is essential for improving maternal and child health outcomes in multiethnic societies.

## Figures and Tables

**Table 1:** Baseline sociodemographic characteristics of the participants (n = 109).

Variable	Category	Frequency (n)	Percentage (%)
Age group	Under 20	19	17.4%
	21–35	75	68.8%
	Over 35	15	13.8%
Marital status	Single	47	43.1%
	Married	28	25.7%
	Free unión	25	22.9%
	Divorced	9	8.3%
Ethnicity	Indigenous	55	50.5%
	Mestizo	51	46.8%
	Afro-Ecuadorian	3	2.7%
Occupation	Housewife	78	71.6%
	Student	9	8.3%
	Employed	21	19.3%
	Studies and Works	1	0.9%
Education level	No formal education	7	6.4%
	Primary	14	12.8%
	Secondary	47	43.1%
	Higher	13	11.9%

**Table 2:** Contingency table of knowledge before and after the intervention (n = 109).

	After: Does Not Know	After: Knows	Total
Before: Does Not Know	44	35	79
Before: Knows	0	30	30
Total	44	65	109

**Table 3:** Differences in knowledge levels before and after the intervention by sociodemographic group (n = 109).

Variable	Category	Knowledge Before (%)	Knowledge After (%)	χ^2^	*p*-Value
Age group	Under 20	31.6	63.2	6.04	0.049 *
	21–35	25.3	58.7		
	Over 35	33.3	60.0		
Marital status	Single	24.2	51.5	8.12	0.043 *
	Married	40.4	66.0		
	Free unión	12.0	64.0		
	Divorced	0.0	25.0		
Ethnicity	Mestizo	37.3	72.5	7.88	0.021 *
	Afro-Ecuadorian	0.0	33.3		
	Indigenous	20.0	49.1		
Occupation	Housewife	25.6	61.5	5.34	0.069
	Student	22.2	66.7		
	Employed	33.3	47.6		
	Studies & Works	100.0	100.0		
Education	No studies	28.6	71.4	10.23	0.017 *
	Primary	7.1	42.9		
	Secondary	36.2	66.0		
	Higher	61.5	84.6		

Note: The asterisk (*) indicates a statistically significant association at the *p* < 0.05 level, based on the chi-square test.

**Table 4:** Main educational promoters supporting breastfeeding knowledge among participants (n = 147 responses).

Educational Promoters	Frequency	Percentage
Healthcare Personnel	49	33.30%
Family Members	41	27.90%
Media	19	12.90%
Women from the Community	18	12.20%
Friends	17	11.60%
Romantic Partner	3	2.00%
Total	147	100.00%

**Table 5:** Cultural beliefs and perceptions associated with breastfeeding abandonment (n = 372 coded mentions).

Attitudes	Frequency	Percentage
Cultural stigmas: shame, return of menstruation, maternal incompetence	117	31.40%
Community and personal pressure: work, studies	98	26.30%
Dietary transition: weaning	61	16.40%
Breast conditions: injuries, inflammation	56	15.10%
Interrupted breastfeeding: vain girls and spoiled boys	40	10.80%

## Data Availability

The research data supporting this publication are available in the OneDrive digital repository “Data” at the following link: https://drive.google.com/file/d/13V2aM6wrDNram7PqCAju-Tnb3L73w3nC/view?usp=drive_link (accessed on 20 January 2024).
